# The complete mitochondrial genome of *Coriandrum sativum*

**DOI:** 10.1080/23802359.2021.1951131

**Published:** 2021-07-15

**Authors:** Yiheng Wang, Qingkuo Lan, Xin Zhao, Luping Wang, Wancong Yu, Bo Wang, Yong Wang

**Affiliations:** Biotechnology Research Institute, Tianjin Academy of Agricultural Sciences, Tianjin, China

**Keywords:** *Coriandrum sativum*, mitochondrial genome, phylogenetic analysis

## Abstract

Using Oxford Nanopore and Illumina sequencing technologies, we reported the first complete mitochondrial genome of the important medicinal and edible plant *Coriandrum sativum*. The complete mitogenome was assembled into two circular-mapping forms of 82,926 bp (cir1) and 224,590 bp (cir2), respectively. There were 28 genes identified in the cir1 mitogenome, which included 14 protein-coding genes, 2 rRNA genes and 12 tRNA genes. There were 62 genes identified in the cir2 mitogenome, which included 41 protein-coding genes, 5 rRNA genes and 16 tRNA genes. Phylogenetic analysis showed that *Coriandrum sativum* was most closely related to *Daucus carota*.

*Coriandrum sativum* (Coriander), a member of the Apiaceae family, belongs to the Mediterranean aromatic herb and is widely cultivated in the world (Tulsani et al. 2020). The coriander leaves, stems and roots with a large number of bioactive compounds of coriander are all edible, which are usually used as vegetable, spice and medicine (Sahib et al. 2013). Samples of *Coriandrum sativum* were obtained from Tianjin Academy of Agricultural Sciences (18°13′47.45″N, 109°30′55.29″E, TAAS) in China. The specimen was deposited at Biotechnology Research Institute of TAAS (voucher number: HB202015). In this study, we used the Oxford Nanopore Technologies (ONT) combined with Illumina Hiseq system to sequence the first complete mitochondrial genome of *C. sativum* (Kan et al. 2020).

The complete mitogenome of *Coriandrum sativum* was assembled into two circular-mapping forms of 82,926 bp (cir1) and 224,590 bp (cir2) in length by the SPAdes assembler (Bankevich et al. 2012), which deposited under the GenBank accession numbers (MW477237 and MW477238). In comparison with other mitogenomes in Apiaceae, the mitogenome of *C. sativum* (∼83kb/∼224kb) was smaller than that of *D. carota* (∼281kb) and *B. falcatum* (∼464kb) (Iorizzo et al. 2012; Kim et al. 2020). The mitochondrial base composition of cir1 and cir2 were A 27.25%, T 27.92%, C 22.66%, G 22.17% and A 27.44%, T 27.41%, C 22.88%, G 22.27%, respectively. There were 28 genes identified in the cir1 mitogenome, which included 14 protein-coding genes, 2 rRNA genes and 12 tRNA genes. There were 62 genes identified in the cir2 mitogenome, which included 41 protein-coding genes, 5 rRNA genes and 16 tRNA genes. Only ten genes contained intron, including eight protein-coding genes (*nad1*, *nad2*, *nad4*, *nad5*, *nad7*, *rps3*, *ccmFc*, and *cox2*), and two tRNA (*trnA-UGC* and *trnP-CGG*). Comparison between the *C. sativum* and *D. carota* mitogenomes, showed that only one unique gene (*ycf2*) was not observed in the latter. The function of *ycf2* gene is unknown, and the similar result appeared in the *Mirabilis himalaica* mitogenome (Yuan et al. 2020).

The phylogenetic tree was constructed by using RAxML (v8.2.9) with 1,000 bootstrap replicates (Stamatakis 2014). A maximum likelihood analysis was performed on nineteen species based on the conserved sequences of the whole mitochondrial genome. The phylogenetic tree showed that *C. sativum* was most related to *D. carota* with 100 bootstrap values (Figure 1). These results will contribute to a better understanding of Apiaceae evolution and further development of molecular markers in *Coriandrum sativum* used for germplasm classification.

**Figure 1.**
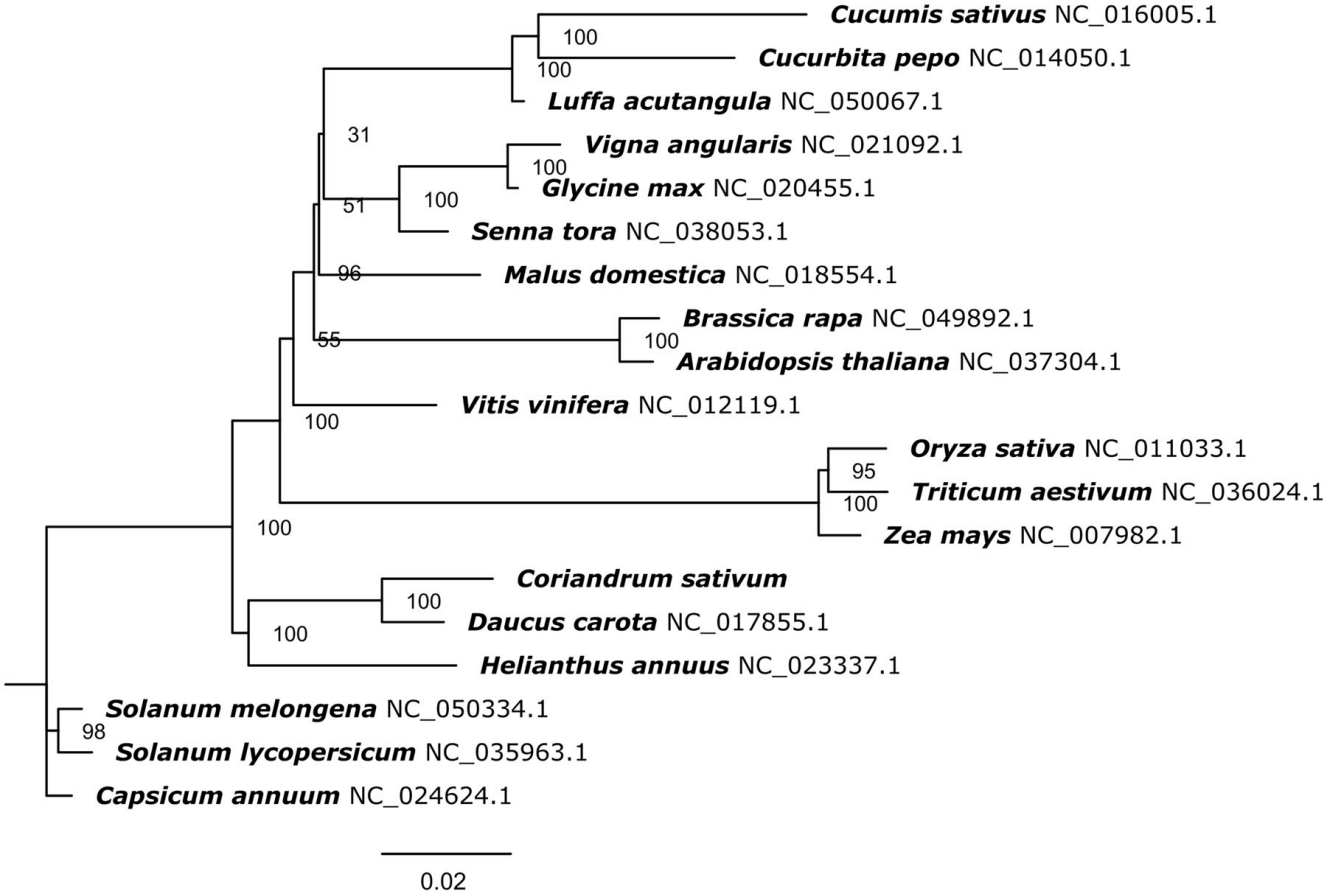
Maximum likelihood tree based on the whole mitochondrial genome from nineteen species. The phylogenetic tree was constructed using RAxML. Numbers on branches were Bootstrap support values (BS).

## Data Availability

The genome sequence data that support the findings of this study are openly available in GenBank of NCBI at [https://www.ncbi.nlm.nih.gov] (https://www.ncbi.nlm.nih.gov/) under the accession no.MW477237-MW477238. The associated BioProject, SRA, and Bio-Sample numbers are PRJNA729412, SUB3752698, and SAMN19115529 respectively.
